# Parathyroidectomy in chronic kidney disease

**DOI:** 10.1590/2175-8239-JBN-2021-S112

**Published:** 2021-12-03

**Authors:** Lillian Andrade da Rocha, Murilo Catafesta das Neves, Fabio Luiz de Menezes Montenegro

**Affiliations:** 1 Universidade Federal de São Paulo, Nephrology Discipline, São Paulo, SP, Brazil.; 2 Universidade Federal de São Paulo, Head and Neck Surgery Discipline, Otorhinolaryngology and Head & Neck Surgery Department, São Paulo, SP, Brazil.; 3 Universidade de São Paulo, Faculdade de Medicina, Central Institute of Hospital das Clínicas, Head and Neck Surgery Division, São Paulo, SP, Brazil.

## 1. Parathyroidectomy indications (PTX)

1.1 Patients with secondary hyperparathyroidism (SHP), with serum PTH level persistently above 800 pg/mL, associated to one or more of the following conditions: 

1.1.1 Hypercalcemia and/or hyperphosphatemia refractory to clinical treatment (Evidence). 

1.1.3 Extraosseous calcifications (soft tissue and/or cardiovascular) or calcific uremic arteriolopathy (calciphylaxis) (Evidence). 

1.1.4 Advanced, progressive and debilitating bone diseases that do not respond to clinical treatment (Evidence). 

1.1.5 Presence of enlarged parathyroid glands on ultrasound (volume > 1.0 cm3) (Opinion). 

1.2 Patients with post-kidney transplant hyperparathyroidism (HPT), when: 

1.2.1 Associated with hypercalcemia of malignancy (total Ca > 14 mg/dL or ionized Ca > 1.80 mmol/L) (Evidence). 

1.2.2 Associated with hypercalcemia and progressive, unexplained loss of graft function (Evidence). 

1.2.3 Persistent hypercalcemia after the first year of kidney transplantation. 

## 2. Preoperative assessment 

2.1 Identify the parathyroid glands by ultrasonography and 99mTc sestamibi scintigraphy whenever possible (Evidence).

2.1.1 The inability or difficulty in performing imaging tests should not delay surgical treatment (Evidence).

2.1.2 If initial surgery fails, a 99mTc sestamibi scintigraphy is recommended to identify ectopic or supernumerary parathyroid glands (Evidence).

2.2 To discard aluminum toxicity in patients with SHP, by means of desferrioxamine test, as directed in the chapter on this topic (Evidence).

2.2.1 In cases of high probability of this association and in the presence of a negative or dubious desferrioxamine test, perform a bone biopsy (Evidence). 

## 3. Types of PTX and intraoperative monitoring

3.1 PTX should be subtotal or total with autograft of parathyroid tissue (Evidence). 

3.1.1 Autograft of parathyroid tissue can be performed in the forearm or in the presternal region (Opinion).

3.2 Additional methods related to PTX, such as intraoperative PTH measurement, freezing and cryopreservation of parathyroid tissue technique could be performed at the surgeon's discretion and according to the availability of the treatment institution. However, its absence should not prevent the performance of PTX (Opinion).

## 4. Treatment of hungry bone syndrome in the immediate postoperative period

4.1 To dose serum calcium (Ca), preferably ionized, at least twice a day until stabilization of its levels and hospital discharge (Opinion). 

4.1.1 In transplant patients or in patients under conservative treatment, monitoring, in addition to Ca, creatinine and magnesium, daily (Opinion). 

4.2 In dialysis patients, introduce IV Ca gluconate immediately after the completion of PTX. Available solutions are 10% calcium gluconate (90 mg of calcium per 10 mL ampoule) and 10% calcium chloride (272 mg of calcium per 10 mL ampoule). Use 10 ampoules of 10% Ca gluconate (or 3 ampoules of 10% Ca chloride) diluted in 400 mL of 0.9% saline solution, infused into a large-bore peripheral vein or central access at a rate of 10 mL/h by means of a continuous infusion pump (or 1mg of elemental Ca/kg/hour). Afterwards, the infusion rate should, based on calcemia, be adjusted every 10 mL/h, every 12 hours, aiming at maintaining serum Ca ≥ 7.5 mg/dL or ionized Ca ≥ 1.0 mmol/L (Opinion).

4.2.1 To provide a supplemental dose of Ca gluconate (one ampoule of IV 10% Ca gluconate, diluted in 50 mL of 5% glucose, in 10-20 minutes) whenever the serum Ca is < 7.5 mg/dL (< 1.0 mmol/L) or the patient presents hypocalcemia symptoms (Opinion). 

4.2.2 Transplant patients may more rarely require intravenous Ca replacement, either continuous infusion or just as a supplement (described earlier), if there are symptoms of hypocalcemia and/or if serum Ca is < 7.5 mg/dL (< 1.0 mmol/L).

4.3 In dialysis patients, initiate Ca carbonate powder or tablet, at an initial dose of 5-15 g (15g = 1 rounded tablespoon), 2 or 3 times a day, after diet release, between meals. In kidney transplant patients, oral replacement should be started after normalization of hypercalcemia, at a dose of 1 g, 2 or 3 times a day (Opinion). 

4.4 To start oral calcitriol at a dose of 2.5 µg/day (in dialysis patients) or 0.75 µg/day (in transplant patients), fractioned in doses concurrent with the use of Ca carbonate (Opinion). 

4.5 After the second postoperative day, Ca carbonate and calcitriol doses should be daily adjusted, according to serum Ca, aiming to suspend Ca gluconate infusion as early as possible (Opinion). 

4.6 In kidney transplant patients, start intensive venous hydration with 0.9% saline solution, at a dose of 2-3 L per day. This procedure could be discontinued after restoring sufficient water intake, with stable renal function. 

4.7 In transplant patients or in patients under conservative treatment, initiate magnesium replacement in case of hypocalcemia-associated hypomagnesemia (Opinion). 

4.7.1 Using 10% magnesium sulfate, 1-2 ampoules, diluted in 5% glucose solution, intravenously, within 1 hour, if magnesium is < 1.2 mg/dL (Opinion). 

4.7.2 Oral replacement should be maintained until normalization of hypocalcemia and hypomagnesemia (Opinion). 

4.8 Discontinue the use of phosphorus binders and calcimimetics. To avoid the use of loop diuretics in transplant patients (Opinion). 

4.9 After PTX, to help manage hypocalcemia during hungry bone, use dialysate with a Ca concentration of 3.5 mEq/L. Hemodialysis should be performed with no heparin for the first 3 days after PTX (Opinion).

4.10 Performing at least one PTH dosage during hospitalization, preferably on the 1st postoperative day (Opinion).

4.11 In dialysis patients, dosing potassium twice a day, during the first 24 hours following PTX, and daily thereafter (Opinion).

## 5. Late postoperative care 

5.1 To monitor serum Ca and P weekly for the first 4 weeks after hospital discharge, and every other week until hungry bone is finished (Opinion).

5.2 To monitor Ca, P, alkaline phosphatase, PTH and 25OH vitamin D every 3 months in the first year after hungry bone has ended. In subsequent years, monitoring should be performed at least every 6 months in dialysis patients, and annually in transplant patients with stable renal function (Opinion).

5.2.1 In dialysis patients, return to the use of phosphorus binders in case of hyperphosphatemia (Evidence). 

5.2.2 Replenish cholecalciferol in case of hypovitaminosis D in accordance with the chapter related to the topic. (Evidence). 

## Rational

SHP is a frequent complication in CKD patients and it requires monitoring and energetic treatment and prevention measures. In case of clinical treatment failure, PTX is the safe surgical treatment with low complication rates and reduced morbidity and mortality in patients with severe hyperparathyroidism^1^ ^-^ ^3^.

Imaging methods for locating parathyroid glands are mostly unable to identify all the hyperfunctioning glands^4^ ^-^ ^6^. Parathyroid ultrasonography and scintigraphy are considered complementary methods, and the difficulty in performing them should not delay surgical treatment, since prepared teams are able to achieve surgical success in most cases^5^. Imaging exams are particularly important in the localization of ectopic glands, which only occur in about 2.5% of patients^5^, or in cases of reoperation due to recurrence or persistence, increasing surgical resolution rates and decreasing complication rates^6^.

In preoperative preparation, aluminum toxicity must be excluded, as this metal is deposited in the bone mineralization front, preventing total bone remodeling that follows PTX. The desferrioxamine (DFO) test has demonstrated high sensitivity and specificity^7^, while bone biopsy is preferred for dubious diagnoses^8^.

Two types of PTX are most commonly performed: subtotal and total with autograft of parathyroid tissue. In subtotal PTX, the surgeon usually elects the smallest gland and/or the one with the best macroscopic appearance as the remaining gland, leaving it whole or performing its partial resection, which is usually identified with non-resorbable sutures to facilitate reinterventions in case of recurrence. In total PTX with autograft, all four glands are removed and a part of the gland with best macroscopic appearance is sectioned and grafted onto a muscle bed, with the most common sites being the forearm and presternal region^9^ ^-^ ^12^.

Studies show that both techniques are effective in controlling HPT. The decision between techniques should take into account clinical and surgical aspects, such as degree of alteration of the parathyroid glands, kidney transplantation possibilities, among others^9^ ^-^ ^14^. The advantages of subtotal PTX are lower rate of severe hypoparathyroidism soon after surgery, since the remaining gland has immediate function, in addition to less need for postoperative calcium and calcitriol replacement. However, studies have observed a higher recurrence rate of HPT, and the re-exploration is more related to surgical complications and morbidities^9^. Total PTX with autograft shows as advantages total removal of all glands from the neck and lower recurrence rate. The latter, when it occurs, is most often due to hyperplasia in the graft topography, and its surgical re-exploration is simpler and with fewer complications. The disadvantages of this technique are the high rate of postoperative hypoparathyroidism and the time lability related to graft functioning, requiring replacement of larger amounts of calcium and calcitriol in the postoperative period^10^.

Intraoperative PTH dosing aims to confirm removal of all hyperfunctional parathyroid glands (total PTX with autograft) or adequate reduction of the hyperfunctional mass (subtotal PTX,) which is possible due to the short half-life of intact PTH^15^ ^-^ ^16^. A 70% or greater decrease among values, collected at baseline and after removal of the glands, predicts surgical success in most patients, with good correlation to long-term PTH values^16^. Although efficient, it can increase the surgical time and rarely has the ability to change the surgical approach, besides being scarcely available in Brazil^5^. A useful alternative is PTH measurement in the first days after PTX, since still elevated PTH levels assume the existence of significant residual parathyroid tissue^16^. 

Intraoperative freezing is the best technique to confirm whether all removed tissues are indeed of parathyroid origin. If not available, the operation could be extended with partial thyroidectomy and thymectomy, if it is suspected that a gland has not been found^17^. 

The auxiliary technique for cryopreservation of parathyroid tissue, for possible future use in cases of definite hypoparathyroidism, was more commonly employed in the past with variable success rates. It demands technical infrastructure with tissue bank requirements^18^. For this reason, and since it is necessary in a minority of patients, its execution has been discontinued in most centers in Brazil ^5^.

After successful PTX, there follows a period known as "hungry bone syndrome", which usually occurs in the first days of postoperative period, but that could also start late, and last up to months. The main characteristics of this phase are hypocalcemia, hypophosphatemia, and elevated alkaline phosphatase^10^ ^,^ ^19^ ^-^ ^21^. An elevated preoperative alkaline phosphatase is the main predictor of more pronounced hypocalcemia in "hungry bone"^19^. At this stage, a major Ca, oral and intravenous, and oral calcitriol replacement is required, and should be started within the first few hours after PTX. It is important to note the risk of phlebitis and necrosis, if extravasation of the solution occurs when administered into a peripheral vein, and reports of hyperchloremic acidosis, with the use of calcium chloride^21^. 

During the "hungry bone" period, it is necessary to pay close attention to serum potassium dosages, as a significant percentage of these patients develop hyperkalemia in the immediate postoperative period, including the need for emergency dialysis. The cause of post-PTX hyperkalemia is controversial, and may be attributed to massive osteoclasts apoptosis and electrolyte balance. Hypocalcemia, resulting from the abrupt reduction in PTH, promotes the influx of sodium (Na) into skeletal muscle cells via a Na-Ca exchange mechanism in the membrane. Then, the entry of intracellular sodium activates the Na/K-ATPase pump, which promotes the efflux of potassium. Preoperative potassium greater than 4.4 mEq/L is a predictor of hyperkalemia in the immediate postoperative period^22^. Dialysis patients are recommended to undergo dialysis in the 24 hours prior to PTX, in addition to dietary potassium restriction in the preoperative period. 

Some patients, especially those with pre-dialysis CKD or transplant recipients, develop hypomagnesemia, which often aggravates sustained hypocalcemia in the postoperative period. Correction of hypomagnesemia is followed by improvement in hypocalcemia. Magnesium replacement is done with intravenous magnesium sulfate or oral magnesium salts until levels return to normal^23^.

The hypophosphatemia that follows PTX is due to the deposition of calcium-associated phosphorus in the matrix mineralization during the process of bone formation. Intravenous phosphorus replacement should be avoided, since it leads to precipitation with calcium. Exception made in the case of severe, symptomatic hypophosphatemia, in which the serum P level is below 1.0 mg/dL^24^. 

In kidney transplant patients, the renal function after PTX may remain stable or be altered, transiently or permanently^25^ ^-^ ^28^. The cause has not yet been fully clarified and it also occurs in patients with primary HPT^26^, possibly related to the hemodynamic effect of calcium and PTH on renal vasculature^27^. Previous tubular lesions and higher PTH values may be related to worsened GFR after PTX ^28^. However, it is clear that persistent post-transplant HPT deteriorates graft function and increases the risk of graft loss^29^, besides the worsening of bone mass and possible implication in the progression of calcification. PTX performed prior to renal transplantation shows better results than if it is performed after it, minimizing the hypercalcemia that sets in after the transplant, resulting from persistent HPT^29^.

After hospital discharge, frequent monitoring of bone metabolism-related biochemical and hormonal parameters is essential to guide dosage adjustments for oral Ca and calcitriol. The need to switch from oral Ca as supplement to its binder function, or even an association of both, should always be considered, in the event of hyperphosphatemia, which should include dietary restriction, use of Ca-free binders if necessary, and readjustment of dialysis dose in parallel. This monitoring aims to prevent recurrence, to act early in case of persistent SHP or even hypoparathyroidism and its consequences^1^ ^,^ ^30^. 

We consider as therapeutic success of surgical treatment when PTH values are reduced to the target range in dialysis patients (2 to 9 times the reference value of the method)^1^ and there is normalization of calcemia in transplant patients with a reduction of PTH > 50% of the baseline value^11^. For kidney transplant patients, the optimal PTH range is variable, and the glomerular filtration rate should always be considered. 
